# The Use of Digital Platforms for Community-Based Monitoring

**DOI:** 10.1093/biosci/biaa162

**Published:** 2021-04-28

**Authors:** Noor Johnson, Matthew L Druckenmiller, Finn Danielsen, Peter L Pulsifer

**Affiliations:** Cooperative Institute in Environmental Sciences, University of Colorado Boulder, Boulder, Colorado, United States; Cooperative Institute in Environmental Sciences, University of Colorado Boulder, Boulder, Colorado, United States; Nordic Foundation for Development and Ecology, Copenhagen, Denmark; director of the Geomatics and Cartographic Research Centre, Carleton University, Ottawa, Ontario, Canada

**Keywords:** digital technology, data, Indigenous and local knowledge, environmental observing, citizen science

## Abstract

Environmental observing programs that are based on Indigenous and local knowledge increasingly use digital technologies. Digital platforms may improve data management in community-based monitoring (CBM) programs, but little is known about how their use translates into tangible results. Drawing on published literature and a survey of 18 platforms, we examine why and how digital platforms are used in CBM programs and illuminate potential challenges and opportunities. Digital platforms make it easy to collect, archive, and share CBM data, facilitate data use, and support understanding larger-scale environmental patterns through interlinking with other platforms. Digital platforms, however, also introduce new challenges, with implications for the sustainability of CBM programs and communities’ abilities to maintain control of their own data. We expect that increased data access and strengthened technical capacity will create further demand within many communities for ethically developed platforms that aid in both local and larger-scale decision-making.

There is rapidly growing interest in community-based monitoring (CBM) of the environment (Conrad and Hilchey 2011, Kouril et al. 2016, Brofeldt et al. 2018), with many CBM programs initiated to equip communities with better information for community decision-making (Wilson et al. 2018). CBM is “a process of routinely observing environmental or social phenomena, or both, that is led and undertaken by community members and can involve external collaboration and support of visiting researchers and government agencies” (Johnson et al. 2015). Danielsen and colleagues have developed a typology of participation in monitoring, ranging from externally driven, professionally executed to autonomous local monitoring programs that have no involvement of professional scientists (Danielsen et al. 2009, 2021 [in this issue]). In contrast to contributory citizen-science approaches, which are usually designed by scientists and involve citizens solely in data collection (Shirk et al. 2012), CBM programs are often informed by community information needs and goals and co-created approaches. In order for CBM programs to inform decisions, their data must be accessible and available in usable formats, making data management a critical component of CBM systems. Increasingly, CBM programs are turning to digital data management systems to facilitate broader and more efficient data access, as well as synthesis and long-term preservation of data.

Within CBM program infrastructure, digital platforms are combinations of hardware and software intended to aid in collecting, archiving, sharing, and using data ­(figure 1) for local or larger-scale assessment, planning, and decision-making. Digital platforms may also create possibilities for interlinking with other data platforms (Pulsifer et al. 2012, Eicken et al. 2014) and support data exchanges between different user groups, such as community residents, scientists, and nonlocal decision-makers (http://stephane-castellani.com/everything-you-need-to-know-about-digital-platforms). CBM platforms act as boundary objects that mediate between different cultures or communities (Star and Griesemer 1989, Pulsifer et al. 2011). At a technical level, platforms can process and transform data to create meaningful information products and representations for different users (Pulsifer and Taylor 2005, Thanos 2014, Pulsifer et al. 2020). Digital platforms therefore potentially present a range of innovations that improve CBM data management.

**Figure 1.**
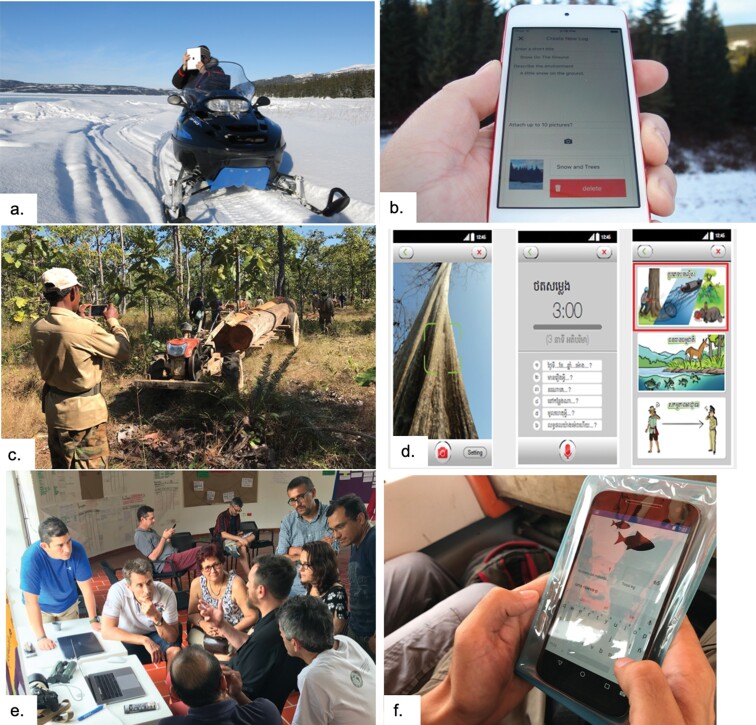
From the Arctic to the forests of Cambodia to the Amazon, digital platforms including smart phone apps facilitate collection and sharing of community observations. (a) Monitoring the land in Nunatsiavut, Labrador, an observer takes a picture with the eNuk app on his iPad. Photograph: Ashlee Cunsolo. (b) The eNuk app on a cell phone. Photograph: Charlie Flowers. (c) A community patrol from Prey Lang documents an illegal timber harvest using the It's Our Forest Too app. Photograph: Ida Theilade. (d) The It's Our Forest Too app allows observers to take photos, record audio, and select from pre given categories such as observations of illegal activities, observations of wildlife, and interactions with officials and offenders. (e) Participants in a design workshop for the Ictio app, which collects observations of fish in the Amazon basin. (f) the Ictio app in action. Photograph: Gina Leite.

The use of digital platforms for CBM is part of a larger transformation in environmental research and monitoring (Hey et al. 2009, Cieslik et al. 2018). The introduction of sensor-based innovations in environmental data collection has been variously dubbed *sensor web*, *digital Earth*, and *smart Earth* (Liang et al. 2005, Hart and Martinez 2015, Gabrys 2016, Bakker and Ritts 2018). These terms refer to the system of sensors and digital infrastructures that capture, store, and share large amounts of continuously collected environmental data (Baker and Millerand 2007). Digital devices, especially smartphone enabled apps, contribute to the development of *citizen sensing*—the involvement of citizens in environmental sensing activities—as a growing subfield of citizen science (Goodchild 2007, Newman et al. 2012, Arts et al. 2015, Cooper 2016, Brenton et al. 2018, Brofeldt et al. 2018, Mazumdar et al. 2019).

Digital platforms may also bring new challenges to the practice of CBM. CBM programs collect and share data from different knowledge systems, including Indigenous knowledge (knowledge held by individuals and communities that identify as Indigenous peoples; for a detailed definition, see ICC 2019, www.inuitcircumpolar.com/icc-activities/ environment-sustainable-development/indigenous-knowledge and the glossary in Eicken et al. 2021 [in this issue], Alessa et al. 2015), local knowledge (knowledge held by residents who engage regularly with the environment and make their own observations on the basis of this engagement; see the glossary in Eicken et al. 2021, Tengö et al. 2021 [in this issue]), and conventional science. When collected and maintained by community members at the local level, each of these types of data can be considered *community data*, a term that reflects community investment in and ownership of data (Pulsifer et al. 2012). Digital management of data from these diverse systems can create challenges for maintaining community control over sensitive data and ensuring local accessibility. Digital platforms may also increase inequities across communities because being able to use digital tools requires technical capacity that may or may not exist at the community level. Moreover, the added program costs of digital platforms may exacerbate challenges of sustaining funding support for CBM programs.

Although the use of digital technologies for collection and management of CBM data has grown, there has been minimal analysis of the implications of this growth for CBM practice or for the use of CBM data for environmental management and decision-making. In the present article, we address the question: *What is the current role of digital platforms in managing community-based monitoring data?*

Conversations during experience-exchange workshops with CBM practitioners have revealed widespread concerns that investments in platforms were being made that may be failing to learn from previous initiatives (Fidel et al. 2017, Johnson et al. 2018). In the present article, we explore digital platforms from a CBM perspective; we examine what they are used for and how and propose strategies for maximizing the benefits of their adoption.

## Survey of CBM programs that use digital platforms

To ascertain the state of digital platform use in CBM programs, we performed a literature review and conducted a survey with CBM practitioners. For the literature review, we searched the databases OneSearch, ProQuest, Web of Science, EBSCOHost, and Google Scholar using the search terms *digital* and *technology* paired with *community-based monitoring*, *participatory monitoring*, and *citizen science* in different combinations. The search results that did not address technology related to CBM or citizen science data management were excluded. We identified 29 articles with strong relevance to digital platforms and CBM, which were reviewed for key themes. These results informed the framing of the present article, including how we assessed challenges and benefits for using digital platforms.

For our survey of CBM programs, we used an online questionnaire to obtain a general understanding of how CBM programs use digital platforms to store and share data. The survey consisted of a combination of open (10) and closed (24) questions, with an option to provide comments to add context for closed questions. The questions were focused on technical aspects of platforms, platform functions and external limitations to functionality, and goals and questions about participation, functionality, and representation of different knowledge types.

Requests for participation in the survey were sent to 28 CBM programs. Respondents were identified using existing formal and informal networks, including the Atlas of Community-Based Monitoring in a Changing Arctic (an online database of CBM programs in the Arctic), CitSci.org (a global database of citizen science programs), and through the authors’ professional networks. We obtained 18 survey responses, including from researchers and program staff affiliated with specific CBM programs (*n* = 10), staff of larger conservation and environmental monitoring initiatives that support CBM programming (*n* = 6), and individuals who have developed platforms to support data sharing by CBM programs (*n* = 2; see the supplemental material). The 10 invited programs that did not participate were geographically diverse and managed by organizations of different sizes, suggesting that their lack of participation did not lead to a sample bias; two of the programs that did not participate replied that they were not using digital platforms.

The respondents used a diversity of platform types, including platforms developed for broad data management (i.e., not specifically for CBM), platforms that were developed for use by multiple CBM programs, and platforms developed for a specific program (table 1). The respondents were geographically broad in distribution; they included programs from the global scale (*n* = 3), the forested regions in the Americas, Madagascar, and Cambodia (*n* = 8), and the Alaskan, Canadian, and Greenlandic Arctic and sub-Arctic (*n* = 7; table 1).

**Table 1.** Summary of our data set of 18 community-based environmental monitoring programs and their data management platforms.

Reference number	Name	URL	Region or Country	Overall framework	Degree of integration between monitoring activity and platform	Interoperable with other systems	Metadata in data discovery catalogues?	Data shared with repositories?
1	Fish Forever	http://data.world	NA	CBR	Partial	Full	Plan	No
2	Community-Based Carbon and Biodiversity Monitoring		Amazon	CS, CBR, CLR	Partial	Full	No	No
3	CitSci.org	www.citsci.org	N/A	CBR, CLR	Full	Part	Plan	Yes^a^
4	SIKU	https://siku.org	Canadian Arctic	CS, CBR, CLR	Full	Part	Yes	Plan
5	DataStream	mackenziedatastream.ca, atlanticdatastream.ca, lakewinnipegdatastream.ca	Canada	CS, CBR, CLR	None	Part	Plan	No
6	BeringWatch Sentinel Program	www.beringwatch.net	Bering Sea, Alaska	CS, CBR, CLR	Full	Part	Plan	No
7	GOAL		Latin America, Caribbean	^b^	None	Part	No	No
8	Programa de Monitoreo Comunitario de Aves de la CONABIO averaves	https://ebird.org/averaves	Mexico	CS, CBR	None	Full	Yes	Yes^c^
9	Citizen Science for the Amazon (Ictio)	Ictio.org	Amazon Basin	CS, CBR, CLR	Full	Part	Plan	Plan
10	Durrell Wildlife Conservation	http://smartconservationtools.org	Madagascar	CS, CBR, CLR	Full	Part	Plan	Plan
11	It's Our Forest Too	https://preylang.net	Cambodia	CBR, CLR	Full	No	No	No
12	Local Environmental Observer Network (LEO)	www.leonetwork.org	N/A but began in Alaska	^d^	Full	Full	Plan	Plan
13	Sea Ice for Walrus Outlook	www.arcus.org/siwo	Bering and Chukchi Seas, Alaska	CBR	Partial	N/A	No	No
14	eNuk	https://enuk.ca	Nunatsiavut, Canada	CS, CBR, CLR	Full	Part	No	No
15	Alaska Arctic Observatory and Knowledge Hub (AAOKH)	https://eloka-arctic.org/sizonet	Alaska Arctic	CS, CBR	None	Part	No	No
16	PISUNA	https://eloka-arctic.org/pisuna-net	Greenland	CLR	None	Part	Plan	Plan
17	Instituto Chico Mendes de Conservação da Biodiversidade		Brazil	CS, CBR	Partial	Full	Plan	Plan
18	WCS Brazil		Brazilian Amazon	CS, CBR	–	–	Plan	Plan

*Note:* The table is organized on the basis of type of platform used: broad data management platforms (1–2), CBM specific platforms developed for use by multiple programs (3–11), and program specific platforms (12–18).

*Abbreviations:* CS, citizen science; CBR, community-based research; CLR, community led research.

a Scistarter.

b “To facilitate community engagement.”

c Global Biodiversity Information Facility, Sistema Nacional de Información sobre Biodiversidad de México, CONABIO (Comisión Nacional para el Conocimiento y Uso de la Biodiversidad).

d Community-based observation of environmental change.

Data from the survey was analyzed using Microsoft Excel. Closed questions were aggregated and open questions and comments were reviewed for context and additional information. Each platform's responses were also reviewed individually to gain greater contextual awareness of platform development, allowing us to draw specific examples into the discussion throughout the present article.

## Survey results: Why and how are digital platforms used in CBM programs?

We have summarized the survey responses across the ­following topics pertaining to how CBM programs use digital platforms: platform goals, intended users, data themes and formats, software and customization, platform functions, timescale of data delivery, role of community members, and approach to sustainability.

### Platform goals

CBM programs may collect data to inform scientific monitoring (e.g., *contributory citizen science* programs *sensu* Shirk et al. 2012), to support community-based research goals, or to support community-led research (which Shirk and colleagues 2012 referred to as *collegial* programs). Our results suggest that many programs aim to contribute to more than one, and often all three, of these approaches (table 1). Community-based research is distinguishable from community-led research as the former is seen as involving both scientists and community members, but often developed with significant input from scientists. Within community-led research, community members shape the goals, methods, and the use of the data or findings from research and monitoring programs.

**Figure 2.**
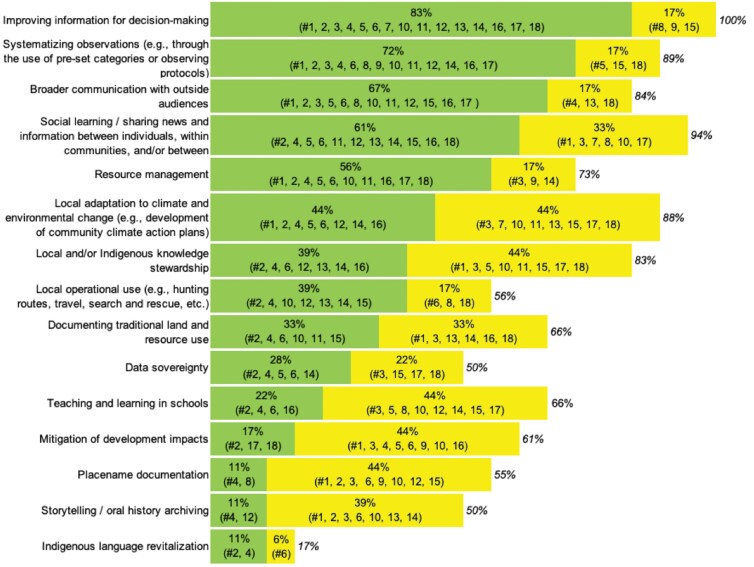
Primary goals (green) and secondary goals (yellow) supported by platform development. Numbers correspond to list of platforms in table 1.

CBM programs select or design digital platforms on the basis of data management goals. These goals usually include sharing information with certain groups of users, such as community members, scientists, or decision-makers. For our survey, we developed a list of 15 potential goals informed by our literature review and by our previous knowledge of various CBM platforms, and asked the respondents to identify whether each was a primary or secondary goal (figure 2). Although we did not define *primary* and *secondary* in the survey, we interpret *primary* as one of the main goals of the platform that was a deciding factor in platform selection or development and *secondary* as a goal that is somewhat less significant to the choice of platform but nevertheless an area in which platform use may benefit the program. On average, platforms listed six primary and four secondary goals, with some listing additional goals in the comments section.

The top five most common goals were improving information for decision-making (100% of the respondents); social learning, or sharing news and information between individuals, within communities, or between communities (94%); systematizing observations (89%); local adaptation to climate and environmental change (88%); and broader communication with outside audiences (84%; figure 2). When noting the improvement of information for decision-making as a goal, 89% of the respondents indicated that they contribute information to observing and decision-making at the global or regional scale, which we defined as any region larger than the local area directly observed by the CBM program.

Many of the top goals as indicated by the survey participants relate to making information accessible, relevant, and usable, reflecting a broader trend in science emphasizing societal relevance, as well as the practical orientation of many CBM programs toward addressing specific information needs. Other goals reflect the unique character of CBM programs that are rooted in community and research priorities. These goals include local or Indigenous knowledge stewardship (83% of the respondents), supporting teaching and learning in schools (66%), storytelling and oral history archiving (50%), and supporting Indigenous language revitalization (17%; figure 2).

### Intended users

**Figure 3.**
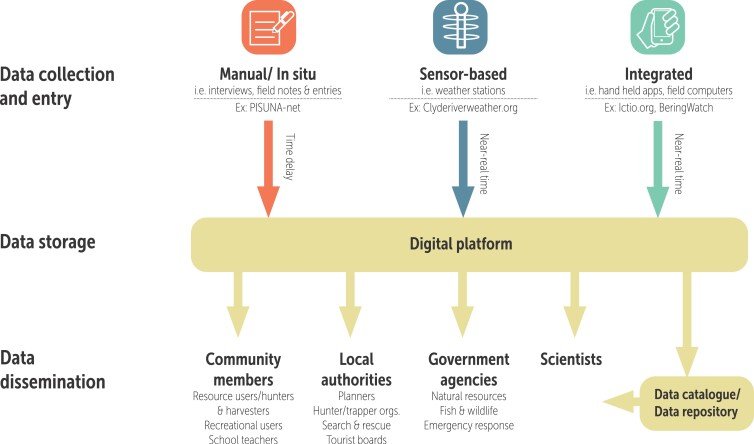
Illustration of digital platforms in CBM programs, the flow of data and the intended users.

CBM platforms aim to make observations easily available, but the intended users of platforms vary. Some are developed for local use or internal use by participants in the monitoring system as a primary goal, whereas others aim to share information broadly with the scientific community or the interested general public (figure 3). All platforms surveyed were designed with the intent of reaching more than one type of end-user group, with an average (mean) of 5.5 intended user groups per platform. Selecting from a predefined list that was developed on the basis of information from the literature review and our previous knowledge of various CBM platforms, the survey respondents identified researchers as the most common primary user (67% of the respondents), followed by local decision-makers (56%), individual community members in general (50%), and renewable resource users (e.g., hunters, fishers; 44%). Three respondents indicated little or no intended platform use at the community or local level. For two of these programs, GOAL and FishForever, CBM platforms were designed primarily for use by program staff of natural resource management or conservation organizations running the CBM program; for the third, Community-Based Carbon and Biodiversity Monitoring, the platform was a simple online database (Microsoft Excel) used by researchers.

Multiple-language support can be a significant factor in determining a platform's accessibility and use. Among the platforms in our survey, the majority were available in English, with Spanish, French, and Portuguese (for those based in Amazonia) as common additional languages. Six of eight Arctic-oriented platforms incorporated Indigenous languages (Inuktitut, Iñupiaq, St. Lawrence Island Yup'ik, North Sami, and Mongolian). Of these, only one included system-functional text in an Indigenous language (meaning that the text of the platform, itself, was translated), whereas four more had plans to translate system text into Indigenous languages in the future. Platforms from South America, Africa, and Asia also hosted (three out of eight) or planned to host (two out of eight) data in Indigenous languages; none of these included or planned to incorporate system functional text in Indigenous languages.

### Data themes and formats

CBM platforms serve as repositories for data related to a wide range of themes or topic areas. The prevalent themes selected in the survey responses, which reflected program-specific goals, included wildlife (sighting, behavior, health, distribution; 72% of the respondents) and wildlife harvesting (61%), other community activities such as boating (61%), seasonality or phenology (e.g., timing of sea ice freeze or thaw, plant and animal life cycle events; 61%), and unusual or anomalous events or observations (e.g., rare wildlife sightings, unusual weather events; 50%).

Surveyed platforms hosted different types of data, including metadata records, video recordings, audio recordings, text records, community-based and non-community-based GIS data, photos or other images, precoded observations (e.g., from a data entry interface with precoded weather descriptors), and *in situ* sensor data, both permanently and periodically deployed. On average, the respondents hosted data in five to six different formats (mean = 5.7).

### Platform software and customization

The survey respondents used different data management platforms depending on their goals and data management needs (table 1). Some adopted general software developed for diverse data management needs; the Community-Based Carbon and Biodiversity Monitoring Program in Amazonia reported using Microsoft Excel and Dropbox to facilitate remote access and data sharing. Others used software programs with more sophisticated options for data visualization. Fish Forever, a program that involves small-scale fishers from 10 countries in collecting catch records, used data.world (http://data.world), a subscription-based platform for data management and visualization. This approach is most practical for sharing with a group of known collaborators because use fees are based on the numbers of users.

Other programs adopted third-party platforms designed to host data from multiple CBM programs. Citsci.org hosts data from a wide range of citizen science projects, including environmental monitoring programs, allowing programs to establish their own projects within the larger site. The Programa de Monitoreo Comunitario de Aves de la CONABIO used aVerAves (https://ebird.org/averaves/home), a regional portal for bird observations based on the eBird platform developed by the Cornell Lab of Ornithology. Both are examples of more general platforms developed by third parties to support data management needs of a range of programs.

Some CBM programs develop their own platforms to store and share observations. These range from a simple, Drupal-based website with an online form for submitting data, such as that maintained by Sea Ice for Walrus Outlook (SIWO; www.arcus.org/siwo), to systems that integrate different technical elements, such as eNuk (https://enuk.ca), a health and environment monitoring application developed with and for the community of Rigolet, Nunatsiavut, Canada (figure 1). eNuk maintains a website and Android and iOS apps used to collect data such as photos, videos, and text descriptions of observations of environmental change from community members.

Platforms successfully developed for use by a specific CBM or observer program may use open source approaches that allow other programs to adopt them with different degrees of modification. The SIZONet platform, which hosts observations related to sea ice and sea ice use from northern coastal Alaska, was adapted for use by the PISUNA (Piniakkanik Sumiiffinni Nalunaarsuineq) program in Greenland, which collects data relevant to natural resource management. Although both platforms are updated and maintained by the same third party—the Exchange for Local Observations and Knowledge of the Arctic (ELOKA)—they are hosted separately and each has its own development process based on program and platform user priorities.

Some programs use multiple platforms to host and share different data sets or to reach different end users. Facebook and other social media platforms are frequently used within communities to share information relevant to environmental and social observing (Danielsen et al. 2017). Some CBM programs have created pages on Facebook to encourage sharing of observations. SIWO, for example, noted that observers preferred to use the program's Facebook page to share information rather than using the data entry form on the SIWO website.

### Platform functions

CBM digital infrastructure can be designed to support different data management functions, including data collection and entry, storage, processing, and dissemination (figure 3). Although some platforms allow data to be entered manually, increasingly, digital devices such as apps for smartphones or custom handheld computers are used for data collection. Although early platforms made use of specially adapted handheld computers (e.g., Gearheard et al. 2011, NWMB 2013), as smartphone designs improve, CBM programs are increasingly using off-the-shelf devices (Oviedo and Bursztyn 2017). Some platforms also collect data automatically from *in situ* sensors. The BeringWatch platform uses iOS or Android mobile apps to collect data about wildlife species and environmental conditions; data are uploaded to their online database for long-term storage, quality control, and reporting. Ictio uses an app that is fully integrated with its online database while allowing bulk data upload so that monitoring data collected independently can be easily shared (figure 1).

Data storage and sharing are essential functions of most CBM platforms. Some store only metadata, pointing users to other sources in which data are held. Others share data but are not the primary repository; data may be provided by another platform via a live web service feed on the basis of a data sharing agreement. Other platforms host data but limit access to certain users, such as those directly involved in the project, providing data summaries or limited data sets to members of the public. Among platforms that participated in our survey, more than half restricted access to at least some data (56% of the respondents), and two-thirds required that data users agree to specific protocols prior to gaining access (67%).

Interoperability—the properties of data and information systems that allow them to interact and share with other information products or systems—is a key feature that facilitates sharing among digital platforms. Ten respondents reported that their platforms were “partially interoperable” and five more were “fully interoperable” (table 1). Although the distinction between full and partial interoperability was left open to interpretation by the survey respondents, many provided additional information. Three platforms mentioned development of an application programming interface (API) to facilitate direct data sharing between systems. An API defines how different types of software interact with one another; it can create a structure for direct sharing of data between digital platforms. Four platforms mentioned allowing data export into csv or GIS shapefiles for import into other systems, which is a more indirect way of sharing data.

For some CBM platforms, making data discoverable means making it findable and accessible by members of a closed, predefined network, such as a nonprofit or government agency. Others, particularly those with links to the scientific community, share metadata (data about data) with data catalogues or deposit data in repositories such as the NSF Arctic Data Center or the Global Biodiversity Information Facility (Chandler et al. 2017). Of our survey respondents, only one shared metadata with data catalogues, a second shared data with a repository, and a third shared with both (table 1). As one respondent explained, “We place a high level of importance on data ownership by participating tribes and any data sharing is solely a matter of tribal discretion.” However, more than half (56%) indicated plans to contribute to data discovery catalogues and six planned to contribute to repositories in the future.

### Time scale

Some CBM programs use sensors and handheld apps capable of facilitating delivery of near-real-time information. Clyderiverweather.org, for example, is a platform that delivers near-real-time weather information from five weather stations near the community of Clyde River, Nunavut, Canada. Other platforms require data to be manually entered into a database and uploaded, delaying availability of information (figure 3). However, not all users need real-time data; accessing data on a periodic set schedule (e.g., weekly or monthly) may be sufficient depending on the use context. For example, Durrell Wildlife Conservation Madagascar, which monitors wildlife poaching, enters data into the SMART platform on a weekly basis. The goal is to provide data access on a timescale in which government officials can use it to intervene into illegal activities. For the Alaska Arctic Observatory and Knowledge Hub (AAOKH), which collects observations about Alaska sea ice, wildlife, and coastal waters, observing changes on the time scale of seasonal cycles is the primary goal. In some instances, CBM programs that focus on seasonal or longer-term scales still see value in real-time communication and exchange of information. BeringWatch, for example, is planning to build a push-notification system into their mobile apps to facilitate real-time comparison of data on storm intensity and activity collected by community observers with remote sensing data collected by the US National Weather Service.

### The role of community members and Indigenous and local knowledge

We asked the survey respondents about the role of community members in platform design, development, data collection, data entry, and platform maintenance. Their responses indicated that community member roles are often limited because of technical and social constraints, with the most common role being data collection or data entry, followed by consulting during platform design or testing. Among the survey respondents, only eNuk indicated that community members play a role in the technical maintenance of the platform.

Internet access is one factor that may constrain the role of community members. Nearly all of the survey respondents (94%) noted that limited Internet access posed challenges for platform use by community members. Half of the respondents reported either “significant” or “very significant” access limitations. In addition, although knowledge coproduction is an increasingly common framework for research (Behe and Daniel 2018, Djenontin and Meadow 2018), coproduction of technology that involves community members as codesigners of these tools is not yet a widely shared norm. Finally, platform development and maintenance require technical skills that can be difficult to come by in remote communities.

We asked the respondents to identify the approximate percentage of data hosted by their CBM platforms that was representative of Indigenous, local, and scientific knowledge, recognizing that there is hybridity between knowledge systems. Nearly all of the platforms hosted at least some conventional science data (89%), and some Indigenous knowledge or local knowledge data (89%). We also asked the respondents to describe the types of data based on Indigenous or local knowledge that their CBM platforms hosted; their responses indicated a wide range that include written records, participatory maps, photos, and audio or video recordings of observations of specific phenomena such as sea ice, weather, wildlife harvesting, and fish and wildlife observations.

### Approach to sustainability

CBM platforms draw on diverse sources of funding and support, including funding from public and private sector supporters and in kind and volunteer assistance. Half of the survey respondents indicated that the CBM platforms they used had received public funding, including research grants, whereas 39% had received support from the philanthropic sector. Only two programs reported receiving support from the private sector, and in both cases private sector funding made up 10% or less of total funding. Two additional platforms either receive or plan to use environmental compensation funds. Several respondents were considering ways to diversify and broaden funding sources and support. Strategies included introducing a fee for use or an annual maintenance fee charged to subscribers. Other platforms specifically referenced the importance of broad support for ensuring sustainability. The Instituto Chico Mendes de Conservação da Biodiversidade commented that “the only guarantee [of sustainability] is the public need for the stored information, generating a public demand for continuation.”

With this understanding of why and how digital platforms are used by CBM, we will now turn to the challenges that have been experienced and how they can be overcome.

## Maximizing the benefits of using platforms for community-based monitoring

**Figure 4.**
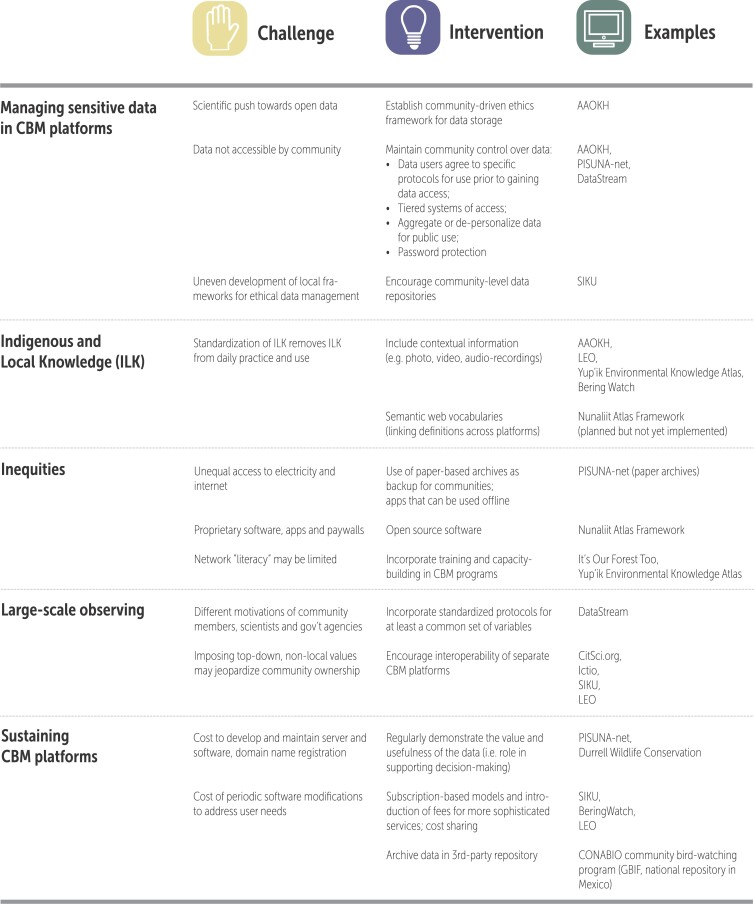
Maximizing the benefits of CBM platform use: Challenges, proposed interventions, and examples.

We have identified five challenges that CBM programs using digital platforms must often navigate: managing sensitive data, incorporating data based on Indigenous and local knowledge, addressing inequities in digital access, contributing to large-scale observing and supporting and sustaining CBM platforms. Below we provide examples from our survey to illustrate how CBM programs address these challenges to maximize the benefits of using digital platforms (summarized in figure 4).

### Managing sensitive data and ensuring community data ownership

Protecting sensitive community data and respecting local data ownership and Indigenous knowledge sovereignty are important aspects of data management for CBM programs. Although there has been a push toward open data within the global scientific community to promote data discovery and use (Williams et al. 2019), many communities want to be able to set limits on data sharing to protect and maintain control over sensitive data (Fidel et al. 2017, Lynn et al. 2019). Open data standards are therefore not always relevant or applicable to data based on Indigenous or local knowledge (RDA-IIDSIG 2019, Tengö et al. 2021 [in this issue]).

Data management innovations exist to address these concerns about data openness (Pulsifer et al. 2012, IASC 2013, Lynn et al. 2019, figure 4). Some platforms provide aggregate data sets for public use, whereas others provide full but depersonalized data. One way to acknowledge community ownership is to require that data users agree to specific protocols for use prior to gaining access to data. These agreements can be built into platforms in different ways. DataStream and AAOKH ask users to agree to use requirements including proper attribution prior to downloading data. In contrast, the SIKU platform, a mobile app and web platform that provides services for ice safety, language preservation, and weather to residents of northern Canada, uses terms of reference to place the responsibility on platform contributors to have data access agreements and licensing in place. Platforms can also create tiered systems of access, with sensitive data password protected to restrict access to a particular subset of users. Lynn and colleagues (2019) recommended that platforms provide options to users to protect or share data at the level of the individual data point, which may encourage collection of data that otherwise might be considered too sensitive.

Data accessibility for community members is also a critical issue for communities; many have experienced a lack of accountability by outside researchers in returning data to the community in a useful format (Gearheard and Shirley 2007). Even in collaborative projects issues can arise, for example, when researchers want to use data for purposes that were not initially discussed with or authorized by the community (Johnson et al. 2018). Intellectual property rights, data sovereignty (recognition that data is subject to governance, including Indigenous self-determination), and customary laws must be respected (Young-Ing 2008, Pulsifer et al. 2012, Scassa et al. 2015). Prior to CBM program data being archived with regional or global repositories, terms of cooperation should be established that address free, prior and informed consent protocols (UN General Assembly 2007, FAO 2016). The CARE principles for Indigenous data governance (collective benefit, authority to control, responsibility, ethics) offer a framework for supporting Indigenous data goals that complements global efforts to advance open data (RDA-IIDSIG 2019).

### Incorporating Indigenous and local knowledge

The formalization of community data in digital platforms revives academic debates about the feasibility and desirability of standardizing Indigenous knowledge for use in environmental management (Nadasdy 1999, Agrawal 2002, Tengö et al. 2021 [in this issue]). Although the use of digital platforms to document Indigenous and local knowledge effectively fixes this knowledge in a context removed from daily practice and use, there have been responsive efforts to develop tools and processes to maintain context, such as the use of narrative formats (e.g., through video or audio recordings; Caquard et al. 2009, Taylor 2013, Aporta et al. 2014, figure 4). Semantic web approaches create knowledge models that map out relationships between terminology and concepts, which can help bridge knowledge systems (Fox and Hendler 2009, Pulsifer et al. 2011, Duerr et al. 2015).

As a related concern, the adoption of digital technologies by community members may result in deskilling, the erosion of practices supported by local and Indigenous knowledge related to travel, hunting, and observation, as well as specific knowledge sets such as taxonomic knowledge (Arts et al. 2015). However, these concerns are often theoretical; research focused on the impacts of technology adoption on local travel, hunting, and harvesting practices suggests that drivers of changes in skill and practice are highly nuanced (Aporta and Higgs 2005), and that it is possible to adapt new technologies in ways that can help maintain Indigenous knowledge systems (Kemper 2015, Zaman et al. 2015).

When data is managed ethically, digital platforms can serve as tools to support Indigenous data sovereignty, ensuring that data is available for local use and under control of local stewards. The Clyde River Knowledge Atlas (clyderiveratlas.ca), for example, was established by Inuit residents of Clyde River, Nunavut, Canada, to ensure that information from research and monitoring conducted in Clyde River was available to residents. Digital mapping platforms for Indigenous land rights, which focus directly on the rights of the involved peoples and communities, may also provide examples of ethical data management practices for the CBM community (box 1).

Box 1. **Land mapping platforms for Indigenous rights.**

*Søren Hvalkof*

Over the past decades, research institutions and NGOs focusing on Indigenous rights have developed web-based, interactive mapping platforms displaying Indigenous territories and local community lands. These platforms have become an important tool in documenting land use patterns and supporting Indigenous and other local communities’ land rights and territorial integrity. While WRI's LandMark mapping initiative (www.wri.org/resources/websites/landmark) focuses on a global scale, many of these platforms have been developed in Latin America, where Indigenous community land rights are most advanced.

The Rainforest Foundation UK's Mapping For Rights (www.mappingforrights.org) is an interactive platform covering the five countries of the Congo Basin; its interactive layers include features such as community mapping, conservation units, concessions and permits, and infrastructure and administrative units. Five access levels range from basic maps to detailed information, with the last one—giving full access to all information and editing—restricted to program staff and community representatives. To control use and misuse, users have to register and gain approval; the user is then granted access to the level of detail needed for her purpose.

Drawing on the success of these participatory mapping platforms, a number of tools have been developed to monitor illegal logging and other infractions by third parties in Indigenous territories. Near-real-time monitoring has been added via mobile devices and satellite technology. Mapping for Rights has launched ForestLink, a real-time CBM tool focusing on illegal logging and deforestation. In Cambodia, the It's Our Forest Too platform similarly focuses on illegal logging and other activities that encroach on the rights of forest dwelling communities (figure 1).

As these platforms focus directly on the rights of the involved communities and peoples, they have developed a legal and normative format that guaranties their involvement in decision making on the flow and the type of data to be displayed. CBM platforms that have placed less emphasis on issues related to community engagement in data management and platform development could therefore benefit from a close examination of these publicly accessible interactive mapping platforms.

### Addressing inequities in digital access

Persistent inequities in power between Western science and governance institutions and Indigenous and local communities shape how digital platforms for CBM are adopted and used (Alexander et al. 2009, Lievrouw 2012) and limit the uptake of CBM data in observing networks that these platforms contribute to (Latham and Williams 2013). Inequality in access to digital devices and supporting infrastructures is of particular concern for CBM platforms. For example, there is a wide variation in Internet bandwidth in different parts of the North American Arctic (Johnson et al. 2018), as well as in Amazonia (survey response). CBM platforms can address these limitations by implementing data collection processes that are independent from Internet access—for example, by allowing mobile apps to be used offline to collect data, which can then be uploaded later when the Internet is available (figure 4).

The use of commercial, subscription-based platforms by CBM programs that charge an annual licensing fee for use can also create access challenges for communities. Even when digital infrastructure is freely provided, this can change abruptly when, for example, data use agreements come to an end or a source of public or private sector support is cut off. Platforms address these concerns in a variety of ways, including through the use of paper-based archives as backup repositories in the event of Internet access failure. The use of open source software, such as the Nunaliit Atlas Framework and CitSci.org, can significantly reduce costs, both for the initial platform setup and for ongoing maintenance and updates.

Being able to successfully use digital tools to manage and access information requires skill and practice. Lack of routine access may lead to differences in competencies needed to successfully participate in digital networks (Lievrouw 2012) and make it more difficult for community members to make use of digital CBM platforms. CBM programs can help raise awareness of the impact of these issues as part of outreach and communication efforts and can promote open dialogue with community members about how to overcome challenges that stem from Internet access limitations.

### Contributing to large-scale observing

There is growing interest in developing regional and global observing systems capable of drawing on diverse sets of observational data, including CBM data. This requires development of interoperable platforms capable of sharing and receiving data from other platforms (Ribes and Bowker 2009). For CBM programs that are primarily interested in supporting local observing for community use, interoperability may seem like a secondary goal to be addressed if and when sharing with other platforms becomes a priority (figure 4). However, there is also growing recognition that capacity to share data between platforms is likely to be useful in the long term (Johnson et al. 2018). For CBM programs, contributing communities must decide which data to share and when to share it. As one survey respondent noted, “the amount of linkage [between our platform and others] will depend on how the community wants to proceed.”

Sharing data to inform regional and global observing and decision-making has technical, scientific, and social requirements. At a technical level, tools exist that allow data sharing in real time as an automated service (such as through an API) or by exporting data from one platform and importing it into another. These tools are most useful when accompanied by metadata that allows users to understand the context surrounding the data.

Practical concerns related to scientific requirements emerge because CBM programs tend to be heterogeneous by design; programs intended to support the information needs of communities and developed through a bottom-up, participatory methodology use a wide range of data collection methodologies (Eicken et al. 2021 [in this issue]). Data standardization, which has proven challenging even among scientific monitoring programs (Millerand and Bowker 2009, Parsons et al. 2011, Yarmey and Baker 2013, Pulsifer et al. 2014), may be undesirable or unachievable for CBM programs on a broad scale. Efforts to standardize data often reflect the priorities of scientists from outside the community and may risk jeopardizing community ownership if done in a way that seems to impose a nonlocal value or goal. At the same time, some CBM programs, such as the Yukon River Inter-Tribal Watershed Council, successfully develop and use a standardized protocol for data collection based on development of shared goals and an understanding that standardization may yield better quality information that can support local decision-making needs (Wilson et al. 2018).

Some academics have raised concerns about the social implications of efforts to draw CBM programs into larger observing networks, particularly when the programs are focused on documenting Indigenous observations (Latham and Williams 2013). These concerns recognize that efforts to coordinate and aggregate observations at larger regional scales have processes of social organization behind them, and that Indigenous peoples are often peripheral to these organizing efforts. As a result, these larger scale efforts are rarely organized to serve information in ways that will benefit Indigenous communities or answer their specific research questions. One way to address this might be to facilitate networking activities among CBM platforms to specifically address how community level priorities and concerns can inform broader scientific, observing, and decision-making efforts.

### Sustaining CBM platforms

Sustaining funding and community support is an ongoing general challenge for CBM programs, which can lead to disruption in data collection and failure to sustain documentation of observing activities over the long term (Johnson et al. 2015, Danielsen et al. 2020, 2021 [in this issue]). Digital data management platforms can exacerbate this challenge when they add to overall program costs ­(figure 4). Developing a novel platform or modifying an existing one can be very costly, and once developed, server and software maintenance and domain name registration create ongoing costs. In addition, periodic software modifications may be needed to address changes in user needs.

The introduction of annual maintenance or use fees can support long-term platform sustainability but must be weighed against the risk of losing potential platform users. Subscription-based models can be tiered to require fees only for more sophisticated services, with some platform elements, such as data access, available free of charge. The SIKU platform, for example, is free to northern residents but has developed payment for service agreements for other CBM programs that are using its services.

Failure to consider the long-term sustainability of CBM platforms brings significant risks that go beyond the loss of the initial investment in infrastructure development, including the disenchantment of community members that have invested social and intellectual capital. If the data management infrastructure fails, community members may lose access to information and data that they depend on. A long-term plan for archiving information in a third-party data repository can mitigate risk.

In spite of these associated costs and risks, CBM platforms have the potential to play a role in program sustainability. Programs that adopt a ready-made platform like CitSci.org may be able to reduce costs for data management while increasing the use of CBM data. The demonstrated use of CBM data in decision-making has been identified as a critical factor in long-term financial and community support (Johnson et al. 2018). When digital platforms make it easier to use CBM data, they can help generate or solidify support. The PISUNA-net platform, for example, was designed to increase support for community-based management of natural resources by delivering relevant information from communities to regional and national decision-makers in Greenland (Danielsen et al. 2020).

## Conclusions

Digital platforms are quickly becoming central to data collection and management for many CBM programs, replacing systems that were much more limited in speed and storage capacity. As we have highlighted, both technical and social challenges have limited the adoption, inclusiveness, and utility of the platforms, but rapid technical development and improvements in remote access are supporting broader deployment and helping to alleviate some issues around access and inequality. Virtual and augmented reality and the use of machine learning create new opportunities for data collection, management and visualization (Striner and Preece 2016).

Moving forward, several critical areas remain to be addressed by CBM programs as well as researchers interested in understanding developments in this growing field. The first is increasing the role of community members in digital platform design, implementation, and use. Two reorientations are needed for this to happen: First, CBM programs need to adopt very strong participatory approaches that emphasize community involvement throughout all stages of program development and implementation. And second, data management needs to become central to the design of CBM programs, rather than an afterthought or something to be addressed only when other program elements are in place. Growing recognition of Indigenous data sovereignty and development of frameworks and protocols for its implementation are also likely to drive a shift in practice toward greater community involvement in CBM platform development and use.

Many platforms are new or still being developed, with ongoing innovation, testing, and experimentation. Although CBM programs remain focused on developing and institutionalizing digital platforms suited to their local needs, we anticipate increasing interest and investment in interoperability, as well as data standardization to support the use of CBM data across scales of decision-making. There is a potential for conflict between the drive for interoperability and standardization and the importance placed on data sovereignty and prioritization of local information needs by community partners. Although there are ways that this tension can be resolved to allow for both community agency and platform interoperability, it remains to be seen whether and how these distinct priorities can be reconciled in a way that prompts a large-scale adoption of interoperability standards and an increased emphasis on data sharing for CBM programs.

As digital platforms become more widely adopted by CBM programs for data collection and management, they are likely to become central to the practice of monitoring and observing. This is a significant reordering of social practice, with a resulting high level of dependency on technology. We have explored some of the issues surrounding this change in the present article, but much more work could be done to examine the tensions that this may create, such as whether or not traditional ways of observing and knowing the environment may be undermined by reliance on digital apps for observational data collection or whether, in contrast, these apps reinforce and facilitate the continuation of place-based ways of observing and knowing the environment. The use of virtual and augmented reality as a means of simulating a direct experience of place for remote platform users, or altering the way that community members experience their local environment as they move through it, will further complicate these questions and is an intriguing area for additional research.

## Supplementary Material

biaa162_Supplemental_File

## Author Biographical

Noor Johnson (Noor.Johnson@colorado.edu) and Matthew Druckenmiller are research scientists at the National Snow and Ice Data Center, at the Cooperative Institute in Environmental Sciences, at the University of Colorado Boulder, in Boulder, Colorado, in the United States. Finn Danielsen is an ecologist with the Nordic Foundation for Development and Ecology in Copenhagen, Denmark. Peter Pulsifer is an associate professor and is the associate director of the Geomatics and Cartographic Research Centre at Carleton University, in Ottawa, Ontario, Canada.
