# Supplementary material for: The Use of Digital Platforms for Community-Based Monitoring
Source: Bioscience. 2021 Apr 28;71(5):452–66. doi: 10.1093/biosci/biaa162 (PMC8106997; doi:10.1093/biosci/biaa162)
Supplement: biaa162_Supplemental_File [file biaa162_supplemental_file.docx]

| **Submission to BioScience -** | |  |  |  |  |  |  |
| --- | --- | --- | --- | --- | --- | --- | --- |
| **Supplementary Materials for manuscript by Johnson et al.:** | | | |  |  |  |  |
| **Digital Platforms for Community-Based Environmental Monitoring** | | | |  |  |  |  |
|  |  |  |  |  |  |  |  |
| **CBM Program name** | **Region** | **Digital platform name and url** | **Overall framework (citizen science = CS; community-based research = CBR; community led research = CLR)** | **Platform designed for single or multiple CBM programs?** | **To what extent is the platform interoperable with other systems?** | **Have metadata been published in data discovery catalogues?** | **Are data shared with any data repositories?** |
| Fish Forever | Global | data.world (http://data.world). | CBR | Designed for broader data management support | Fully interoperable | Not yet, but planned for the future | No |
| CitSci.org | Global | www.citsci.org | CBR; CLR | Multiple | Partially interoperable | Not yet, but plan to contribute to DataONE and GBIF | Yes: scistarter as a repo for project metadata; plan to share data with GBIF |
| Local Environmental Observer (LEO) Network | Global with origins in the Arctic | www.leonetwork.org | Community-based "observing" of environmental change | Single | Fully interoperable | Not yet, but planned for the future; we do show up in some citizen science data discovery catalogs. E.g. US Climate Resilience Tool Kit. | Not yet, but an annual digest for archive is planned. |
| Sea Ice for Walrus Outlook | Northern Bering Sea and Southern Chukchi Sea, Alaska | Sea Ice for Walrus Outlook, https://www.arcus.org/siwo | CBR | Single | Note sure | No | No |
| SIKU | Canadian Arctic | SIKU.org | CS; CBR; CLR | Multiple | Partially interoperable | Yes: Polar Data Catalog | Not yet, but planned for the future |
| eNuk | Nunasiavut, Canada | [https://enuk.eco/.](https://enuk.eco/) | CS; CBR; CLR | Single | Partially interoperable | No | No |
| Alaska Arctic Observatory and Knowledge Hub (AAOKH) | Northern Alaska | https://eloka-arctic.org/sizonet | CS; CBR | Single | Partially interoperable | No | No |
| DataStream | Canada | mackenziedatastream.ca; atlanticdatastream.ca; lakewinnipegdatastream.ca | CS; CBR; CLR | Multiple | Partially interoperable | Not yet, but planned for the future | No |
| BeringWatch Sentinel Program | Bering Sea region of Alaska, with expansion to mainland Alaska and Canada | <https://www.beringwatch.net/> | CS; CBR; CLR | Multiple | Partially interoperable | Not yet, but planned for the future. We have produced FGDC compliant metadata as required by some funding institutions and submitted metadata as required. | No, but there is a dedicated Beringwatch data portal; data sharing practices reflect data ownership by participating tribes and is solely a matter of tribal discretion. |
| PISUNA | Greenland | <https://eloka-arctic.org/pisuna-net/> | CLR | Single | Partially interoperable | Not yet, but planned for the future | Not yet, but planned for the future |
| GOAL in Latin America and the Caribbean | Latin America/Caribbean | https://www.dimagi.com/commcare/pricing/; https://powerbi.microsoft.com/en-us/ | "To support the delivery of GOAL's programs and facilitate community engagement" | Multiple | Partially interoperable | No | No |
| Programa de Monitoreo Comunitario de Aves de la CONABIO | Mexico | averaves (https://ebird.org/averaves/home) | CS; CBR | Multiple | Fully interoperable | Yes: SNIB of CONABIO, Mexico | Yes: GBIF, SNIB (CONABIO, Mexico) |
| Instituto Chico Mendes de Conservação da Biodiversidade | Brazil | BD Monitora | CS; CBR | Single, integrated monitoring program (CBM one component) | Fully interoperable | Not yet, but planned for the future | Not yet, but planned for the future |
| WCS Brazil | Brazilian Amazon | Platform in early development | CS; CBR | Single | Note sure | Not yet, but planned for the future | Not yet, but planned for the future |
| Citizen Science for the Amazon | Amazon Basin | Ictio.org | CS; CBR; CLR | Multiple | Partially interoperable | Not yet, but planned for the future | Not yet, but planned for the future |
| Community-based carbon and biodiversity monitoring | Amazon | Excel and Dropbox | CS; CBR; CLR | Designed for broader data management support | Fully interoperable | No | No |
| Durrell Wildlife Conservation Madagascar | Madacascar | http://smartconservationtools.org/ | CS; CBR; CLR | Multiple | Partially interoperable | Not yet, but planned for the future | Not yet, but planned for the future |
| It's Our Forest, Too | Cambodia | <https://preylang.net/> | CBR; CLR; Protection of forest resources, advocacy, petition the government to protect the forest and enforce the forest law | Multiple | Very limited in interoperability | No | No |
|  |  |  |  |  |  |  |  |
| **CBM Program name** | **Primary goals** | **Primary (intended) user(s)** | **Role of community members** | **Additional roles** | **Languages** | **Majority of data (>50%) based on conventional science** | **Percent (range) of data based on Indigenous or local knowledge** |
| Fish Forever | Local adaptation to climate and environmental change; Resource management; Systematizing observations; Broader communication with outside audiences; Improving information for decision-making | Staff of organization and key partners | They have no direct role in supporting the platform |  | Not sure | Yes | Indigenous: 21-40%; Local: 1-20% |
| CitSci.org | Systematizing observations; Broader communication with outside audiences; Improving information for decision-making | Individual community members; researchers | Data collection and/or entry; Sharing information about the platform with the community |  | English | Yes | Indigenous: 1-20%; Local: 21-40% |
| Local Environmental Observer (LEO) Network | Storytelling / oral history archiving; Local adaptation to climate and environmental change; Social learning; Local and/or Indigenous knowledge stewardship; Systematizing observations; Local operational use; Broader communication with outside audiences; Improving information for decision-making; Sharing of information between different knowledge systems. | Individual community members; renewable resource users; local decision-makers; topic experts participating in consulting roles. | Consulting role; Data collection and/or entry; Sharing information about the platform with the community | Real time collaboration via webinars and workshops. | English (primary), all Arctic National languages, partial: Yupik, North Sami, French, Spanish, Mongolian, Portuguese. | No | Indigenous: 21-40%; Local: 41-60% |
| Sea Ice for Walrus Outlook | Social learning; Local and/or Indigenous knowledge stewardship; local operational use; Improving information for decision-making; share scientific information in an accessible format for Indigenous communities. | Renewable resource users | Consulting role; Data collection and/or entry; Sharing information about the platform with the community |  | English, with some use of Iñupiaq and St. Lawrence Island Yupik. | No | Indigenous: 41-60%; Local: 1-20% |
| SIKU | Documenting traditional land and resource use; Storytelling / oral history archiving; Local adaptation to climate and environmental change; Resource management; Social learning; Placename documentation; Local and/or Indigenous knowledge stewardship; Data sovereignty; Teaching and learning in schools; Systematizing observations; Local operational use; Broader communication with outside audiences; Improving information for decision-making; Indigenous language revitalization; Inuit self-determination in research | Individual community members; renewable resource users; researchers; local decision-makers; co-management institutions | Consulting role; Data collection and/or entry; Sharing information about the platform with the community |  | English, partial in Inuktitut (ice, wildlife, placenames) but text/navigation not yet completed. Intention to have full language support in the future. | No | Indigenous: 21-40%; Local: 21-40% |
| eNuk | Local adaptation to climate and environmental change; Social learning; Local and/or Indigenous knowledge stewardship; Data sovereignty; Systematizing observations; Local operational use; Improving information for decision-making | Individual community members; renewable resource users; local decision-makers | Consulting role; Technical platform maintenance ;Data collection and/or entry; Sharing information about the platform with the community | The community is leading the development, and will co-analyze and co-interpret findings based on data collected through the tool | English only, for now. | No | Indigenous: 21-40%; Local: 21-40% |
| Alaska Arctic Observatory and Knowledge Hub (AAOKH) | Documenting traditional land and resource use; Social learning; Local operational use; Broader communication with outside audiences; | Researchers | Data collection and/or entry |  | English only, although Iñupiat terms are included for locations, fish, birds, and wildlife and in text comments when provided by observers | No (none) | Indigenous: 21-40%; Local: 41-60% |
| DataStream | Local adaptation to climate and environmental change; Resource management; Social learning; Data sovereignty; Broader communication with outside audiences; Improving information for decision-making; Improving access to data; standardizing water data reporting | Individual community members; researchers; local decision-makers; non-local decision makers; co-management institutions | Consulting role; Data collection and/or entry; Sharing information about the platform with the community; | Providing feedback on usability and performance of the platform to ensure it is meeting community needs | English | Yes (all) | None |
| BeringWatch Sentinel Program | Documenting traditional land and resource use; Local adaptation to climate and environmental change; Resource management; Social learning; Local and/or Indigenous knowledge stewardship; Data sovereignty; Teaching and learning in schools; Systematizing observations; Broader communication with outside audiences; Improving information for decision-making; Invasive species monitoring | Individual community members; renewable resource users; researchers; local decision-makers; co-management institutions | Consulting role; Data collection and/or entry; Sharing information about the platform with the community; Providing funding for platform |  | English (with technical capacity to expand to multiple languages) | Yes | Indigenous: 1-20%; Local: 1-20% |
| PISUNA | Local adaptation to climate and environmental change; Resource management; Social learning; Local and/or Indigenous knowledge stewardship; Teaching and learning in schools; Systematizing observations; Broader communication with outside audiences; Improving information for decision-making | Renewable resource users; researchers; local decision-makers; non-local decision-makers; co-management institutions | Consulting role; Data collection and/or entry; Sharing information about the platform with the community | Using the platform in discussions with government decision-makers at municipal and central level | English and Greenlandic (Translation is only partial) | No (none) | Indigenous: 81-100%; Local: 1 - 20% |
| GOAL in Latin America and the Caribbean | Improving information for decision-making; Adaptation management and accountability for the delivery of development and humanitarian programmes. | Staff of organization are the primary users; data is shared with communities | They have no direct role in supporting the platform |  | English, Spanish, French | No | Indigenous: 1-20%; Local: 61 - 80% |
| Programa de Monitoreo Comunitario de Aves de la CONABIO | Placename documentation; Systematizing observations; Broader communication with outside audiences | Individual community members; researchers | Data collection and/or entry |  | Spanish and English | Yes (all) | None |
| Instituto Chico Mendes de Conservação da Biodiversidade | Mitigation of development impacts; resource management; systematizing observations; broader communication with outside audiences | Researchers; local decision-makers; co-management institutions | Consulting role; Data collection and/or entry; Sharing information about the platform with the community | Interpreting results with local knowledge | Portuguese | Yes | Local: 1-20% |
| WCS Brazil | Mitigation of development impacts; Resource management; Social learning; Improving information for decision-making; establish a basis for management of natural resources (e.g. fisheries, subsistence hunt) | Individual community members; renewable resource users; researchers; local decision-makers; co-management institutions | Consulting role; Data collection and/or entry; Sharing information about the platform with the community |  | Portuguese | No | Indigenous: 21-40%; Local: 21-40% |
| Citizen Science for the Amazon | Systematizing observations; Understanding fish migration and the environmental factors that influence them | Renewable resource users; researchers; local decision-makers; co-management institutions | Consulting role; Data collection and/or entry; Sharing information about the platform with the community |  | English, Spanish, Portuguese | Yes | Indigenous: 21-40%; Local: 21-40% |
| Community-based carbon and biodiversity monitoring | Documenting traditional land and resource use; Mitigation of development impacts; Local adaptation to climate and environmental change; Resource management; Social learning; Local and/or Indigenous knowledge stewardship; Data sovereignty; Teaching and learning in schools; Systematizing observations; Local operational use; Broader communication with outside audiences; Improving information for decision-making; Indigenous language revitalization; Climate Change and Carbon cycle | Researchers | They have no direct role in supporting the platform |  | English | Yes | Indigenous: 1-20% |
| Durrell Wildlife Conservation Madagascar | Documenting traditional land and resource use; Resource management; Systematizing observations; Local operational use; Broader communication with outside audiences; Improving information for decision-making; Quick response by park managers; Gathering evidence for improving law enforcement | Researchers; local decision-makers; co-management institutions | Consulting role; Data collection and/or entry; Sharing information about the platform with the community | Using SMART in their CBM activities, local community members can help civil societies to play their advocacy role to complain with corruption or other unfair activities inside the protected area | French (however all our data are collected through local dialects/languages) | Yes |  |
| It's Our Forest, Too | Documenting traditional land and resource use; Resource management; Social learning; Systematizing observations; Broader communication with outside audiences; Improving information for decision-making; Documenting illegal logging and other forest crimes. | Individual community members; general public (including the media). | Consulting role; Data collection and/or entry; Sharing information about the platform with the community | Initial design of smartphhone app, feed-back and continuous development of app according to community needs, ownership of data and decisions on publication of results, press conferences and dissemination of knowledge on social media | Khmer and English | No | Indigenous: 61-80%; Local: 21-40% |
